# OVERCOMING TECHNOLOGY BARRIERS: A US ENTERPRISE’S JOURNEY IN COVID VACCINE ACCESS FOR OLDER ADULTS IN SOUTHWEST OHIO

**DOI:** 10.1093/geroni/igae098.1580

**Published:** 2024-12-31

**Authors:** Cleopatra Kum, Flavius Kum, Crescentia Kum, Mirabelle Kum

**Affiliations:** University of Cincinnati, Cincinnati, Ohio, United States; Family Support Care LLC, Hamilton, Ohio, United States; KumZ International Medical Foundation, Douala, Littoral, Cameroon; KumZ International Medical Foundation, Douala, Littoral, Cameroon

## Abstract

It is no hidden fact, that mobility and transportation, especially among seniors and people with disabilities can play a significant role in social isolation and access to basic healthcare and other needs. The importance of safe and effective transportation options was highlighted during the COVID 19 pandemic, when government agencies and community partners worked in collaboration to propose innovative solutions, to ease access to vaccines for seniors through reliable transportation. Family Support Care LLC (DBA KumZ Transport), a Medicare certified home healthcare agency in Ohio, answered the call when one of the Area Councils on Aging reached out to its network of providers to seek agencies willing to transport seniors to receive their COVID 19 vaccines. The purpose of this presentation is to discuss the lived experience of Family Support Care LLC with the use of technology while transporting older adults to get their COVID 19 vaccines. Decisions on technology use was a very important part of the planning phase. Because transportation and logistics management were new to the agency, we faced several barriers as we implemented technology in aging transportation. As a family-owned small business, access to resources and manpower were a major challenge. As a result, we relied heavily on transportation technology for improved productivity and reliability. Despite these challenges, we learned and implemented valuable lessons as we collaborated with more transportation brokers. Technology played a vital role for our agency in planning rides for seniors during the COVID 19 pandemic.

